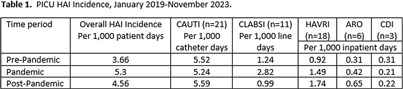# Burden of Healthcare-Associated Infections in a Pediatric Intensive Care Setting Before, During, and After the Pandemic

**DOI:** 10.1017/ash.2024.117

**Published:** 2024-09-16

**Authors:** Meghan Engbretson, Josh Schaffzin, Dayre McNally, George Gubb, Nisha Thampi

**Affiliations:** Children’s Hospital of Eastern Ontario

## Abstract

**Background:** Adult rates of non-COVID-19-related healthcare-associated infection (HAI) initially decreased and subsequently increased during the COVID-19 pandemic. Little is known about pediatric HAI rates during this period. **Methods:** A retrospective review of HAIs was conducted for patients admitted to the intensive care unit (PICU) at a pediatric tertiary care hospital between January 1, 2019 and November 30, 2023. Patients who spent ≥48 hours in the PICU were included. Surgical site infections were excluded. Data were obtained from infection surveillance reports; each HAI was reviewed for validity and attribution based on National Healthcare Safety Network definitions. HAIs were grouped into 3 time periods: pre-pandemic (January 2019-February 2020), pandemic (March 2020- February 2022), and post-pandemic (March 2022-November 2023). Infection rate ratios were calculated for pre-pandemic and post-pandemic periods. **Results:** Among 2,959 PICU patients admitted during the study period, there were 60 HAI events (4.78 per 1,000 patient days). Rates generally remained steady throughout with slight increases and decreases between time periods (Table 1). There was no significant difference in CAUTI, CLABSI, or HAVRI rates noted in the PICU between pre-pandemic and post-pandemic periods despite a significantly higher device utilization ratio in the post-pandemic period for both urinary catheters and central lines (IRR, 0.89; p < 0 .05; 95% CI, 0.82-0.97). The most frequent HAI in all time periods was CAUTI. **Conclusion:** Unlike reports from adult centers, no significant variation between time periods was noted for HAIs in our pediatric center. Despite numerous COVID-19-related changes in infection prevention and control measures and contexts throughout the study period, HAI rates remained stable. This may be due in part to the lower burden of critically ill COVID-19 pediatric patients compared to adult populations. Additionally, this could indicate resiliency and consistency in practice among pediatric providers throughout the pandemic. Further evaluation of pediatric HAIs in the context of the COVID-19 pandemic may reveal practices that could be replicated elsewhere to control HAI rates.

**Figure:**